# Halloysite-TiO_2_ Nanocomposites for Water Treatment: A Review

**DOI:** 10.3390/nano13091578

**Published:** 2023-05-08

**Authors:** Mahmoud Abid, Abdesslem Ben Haj Amara, Mikhael Bechelany

**Affiliations:** 1 Institut Européen des Membranes, IEM, UMR 5635, University Montpellier, ENSCM, CNRS, 34730 Montpellier, France; mahmoud.abid93@gmail.com; 2 Laboratory of Resources, Materials & Ecosystem (RME), Faculty of Sciences of Bizerte, University of Carthage, Bizerte 7021, Tunisia; abdesslem.bha@gmail.com; 3 Gulf University for Science and Technology, GUST, West Mishref, Hawalli 32093, Kuwait

**Keywords:** halloysite, nanocomposite, nanoparticles, nanofibers, electrospinning, photocatalysis and water treatment

## Abstract

Halloysite nanotubes (HNTs) are clay minerals with a tubular structure that can be used for many different applications in place of carbon nanotubes. Indeed, HNTs display low/non-toxicity, are biocompatible, and can be easily prepared. Moreover, the aluminum and silica groups present on HNTs’ inner and outer surfaces facilitate the interaction with various functional agents, such as alkalis, organosilanes, polymers, surfactants, and nanomaterials. This allows the deposition of different materials, for instance, metal and non-metal oxides, on different substrate types. This review article first briefly presents HNTs’ general structure and the various applications described in the last 20 years (e.g., drug delivery, medical implants, and energy storage). Then, it discusses in detail HNT applications for water purification (inorganic and organic pollutants). It focuses particularly on HNT-TiO_2_ composites that are considered very promising photocatalysts due to their high specific surface area and adsorption capacity, large pore volume, good stability, and mechanical features.

## 1. Introduction

Clay is a safe and human-friendly material that has been used in a wide variety of applications for centuries. The current knowledge of clay minerals allows modulating the clay particle size, shape, and surface chemistry to improve its morphological characteristics at the nanoscale [1,2].

Among natural clays, halloysite nanotubes (HNTs) are a particularly interesting nanomaterial due to their biocompatibility and low toxicity, high aspect ratio, empty inner cavity, and different inner and outer surface chemistries [3]. Berthier (1826) was the first to describe halloysite as a 1:1 dioctahedral kaolin clay mineral with octahedral gibbsite Al(OH)_3_ and tetrahedral SiO_4_ layers (i.e., HNT), forming multilayered rolled hollow cylinders [4,5,6]. Layered halloysite mainly occurs in two different polymorphic forms (hydrated form with an interlayer spacing of 10 Å, and anhydrous form with an interlayer spacing of 7 Å) that have the same formula: Al_2_Si_2_O_5_(OH)_4_ [4,5,7,8].

In general, HNTs’ outer and inner diameters and lengths are 50–100 nm, 10–30 nm, and 100–200 nm, respectively. The outer surface is made of Si-O-Si groups, and the inner surface is made of Al-OH groups. The thermal stabilization effect is explained by the entrapment of polymer degradation products in the nanotube lumen.

**Figure 1:**
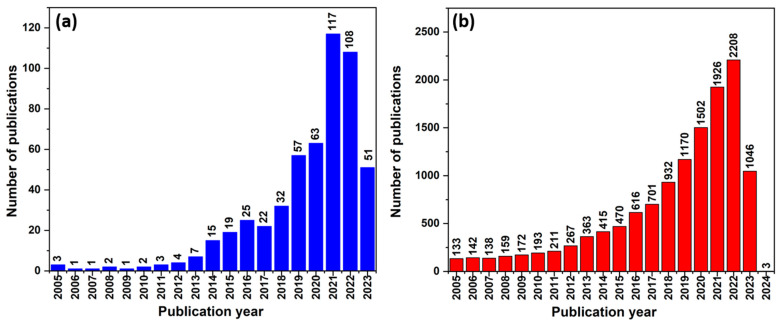
Number of scientific publications per year on (**a**) HNT and (**b**) HNT-TiO_2_ (Science Direct search performed on 21 April 2023).

**Figure 2:**
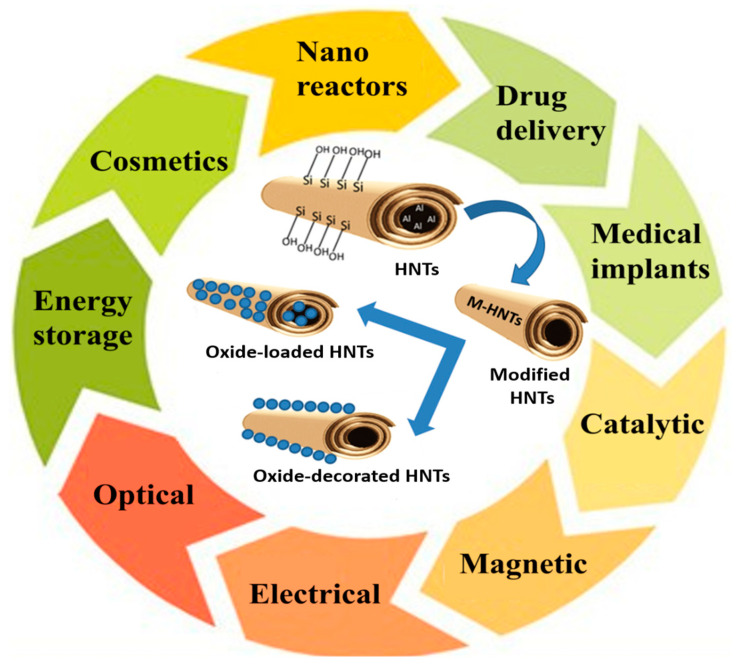
Different applications of HNTs.

The growing interest in HNT is reflected by the increasing number of scientific publications and patents in the last 20 years (Figure 1a). Based on a Science Direct search conducted, it was found that over the last decade, halloysite nanocomposites have received much attention for many applications (Figure 2). Some of these applications include drug delivery [9,10], medical implants [11,12], nanoreactors [13,14], catalysis [15,16,17,18], optics [19,20], electrical engineering [21,22], magnetic materials [7,23], and energy storage [24]. This attention is due to their physicochemical properties, including their tubular structure, ion exchange, and hydrophobicity. Such a variety of applications has been possible because HNTs’ inner lumen and outer surface can be modified with various interface functional compounds. This allows the deposition of different materials, such as oxides, metals, and non-metals, on different substrate types. 

This review article first briefly describes the general structure and the various applications of halloysite nanocomposites that have been proposed in the last 20 years (from drug carriers to polymer fillers). Particularly, halloysite–semiconductor composites are excellent adsorbents, and they can be easily separated from the treated solutions. Such composite systems have a high adsorption capacity and can be used for water purification to remove inorganic and organic pollutants. Much attention has been devoted to the development of inexpensive and environmentally friendly TiO_2_-HNT composites that are very effective catalysts for the degradation of various organic pollutants present in water (e.g., dyes, pesticides, and pharmaceuticals) (Figure 1b). This is due to their excellent photocatalytic activity and mechanical stability. In water treatment, TiO_2_-HNT composites can efficiently degrade various organic pollutants. Additionally, it has been shown that TiO_2_-HNT composites can be used to remove bacteria and viruses from water, thus representing a potential alternative to traditional water disinfection methods. The enhanced photocatalytic activity of TiO_2_-HNT composites is explained by the synergistic effect of TiO_2_ and HNT [25]. 

TiO_2_-HNT composites for water treatment can be synthesized using various methods, including sol–gel, hydrothermal, and in situ deposition. These methods allow for precisely controlling the composite structure, morphology, and properties that can influence photocatalytic activity. 

Overall, TiO_2_-HNT composites display great potential for water treatment, and consequently, the number of publications on this topic is expected to steadily increase in the coming years. Researchers are working to optimize their properties and assess their viability in practical settings.

## 2. Halloysite Structure and Characterization

Halloysite (Al_2_(OH)_4_Si_2_O_5_.nH_2_O) has a 1:1 dioctahedral aluminosilicate clay mineral structure and belongs to the kaolinite group. It is classified as a “nanomaterial” due to its size. Halloysite is mainly in two forms: hydrated and dehydrated. The hydrated form (i.e., halloysite 10 Å) occurs when *n* = 2 water molecules are present between the nanotube multilayers. Upon heating or in vacuum conditions, water molecules are removed (i.e., n = 0), thus obtaining the dehydrated form (i.e., metahalloysite or halloysite 7 Å) [26,27]. 

Halloysite particles display many different morphologies, particularly elongated tubules and short tubular, spherical, and plate-like particles [28,29,30]. This variability is explained by its crystal structure, degree of alteration, chemical composition, and degree of dehydration. Moreover, platy forms are relatively rich in Fe, unlike tubular particles. In addition, in tubular particles, the tube length is negatively affected by replacing Fe^3+^ with Al^3+^ [28].

Halloysite contains impurities of other minerals, such as iron and titanium oxides, potassium, sodium, calcium, and magnesium cations, and organic substances that vary in function depending on the mining site. The HNT structure is described as a monoclinic unit cell with a = 5.14, b = 8.9, c = 7.25–20.7 Å, α = 97–104°, β = 90–91.8°, γ = 90° [28]. The specific surface area (SSA) normally ranges between 50 and 60 m^2^/g and rarely is >100 m^2^/g. HNT’s pore size distribution ranges between 2 and 50 nm, which makes it suitable for various applications, especially as a nanoscale matrix. Halloysite’s cation exchange capacity varies between 20 and 60 cmol/kg depending on factors such as its purity, particle size, and micro-topography [28].

**Figure 3:**
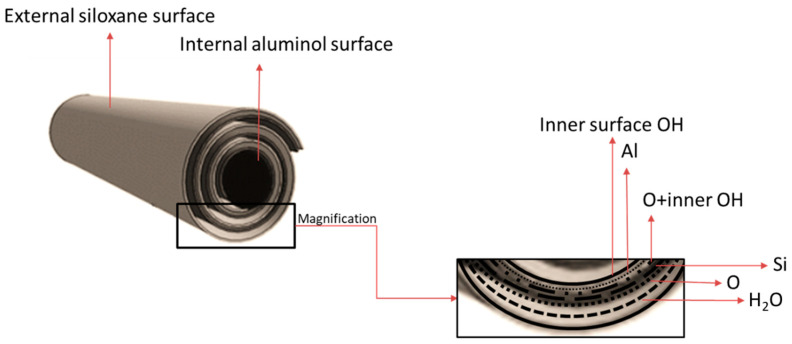
Schematic structure of halloysite.

The HNT tubular structure is the result of the difference between the larger tetrahedral silicate layer (SiO_2_) and the smaller octahedral gibbsite layer (Al_2_O_3_). HNTs with an outer diameter of 50–70 mm, an inner lumen diameter of 15 nm, and a length of ~0.5–1 μm are chemically stable. Generally, HNT length is >30 μm [7], and the outer and inner diameters are 30–190 nm and 10–100 nm, respectively. The outer layer mainly consists of siloxane groups (Si-O-Si), along with aluminol (Al-OH) and silanol (Si-OH) groups, whereas the inner layer is composed only of aluminol groups and is positively charged (Figure 3) [31]. The outer surface is hydrophilic, and the inner cavity is hydrophobic [32]. HNT can absorb and release hydrophilic and hydrophobic compounds [33]. Due to its active surface and shape, HNT can easily support cationic compounds [34]. HNTs display very good mechanical properties and are therefore used to increase the strength of many polymer nanocomposites. The porous surface with high total pore volume (TPV) and SSA allows modifying the HNT surface with various materials. However, TPV and SSA can vary, mainly depending on the level of impurities present in the nanotubes [35,36]. HNT physicochemical features and the possibility of surface modifications can increase the nanotube’s surface resistance.

In practice, HNTs are considered promising nanomaterials due to their biocompatibility, non-toxicity, good dispersion, and large SSA, which improves their performance [33].

**Figure 4:**
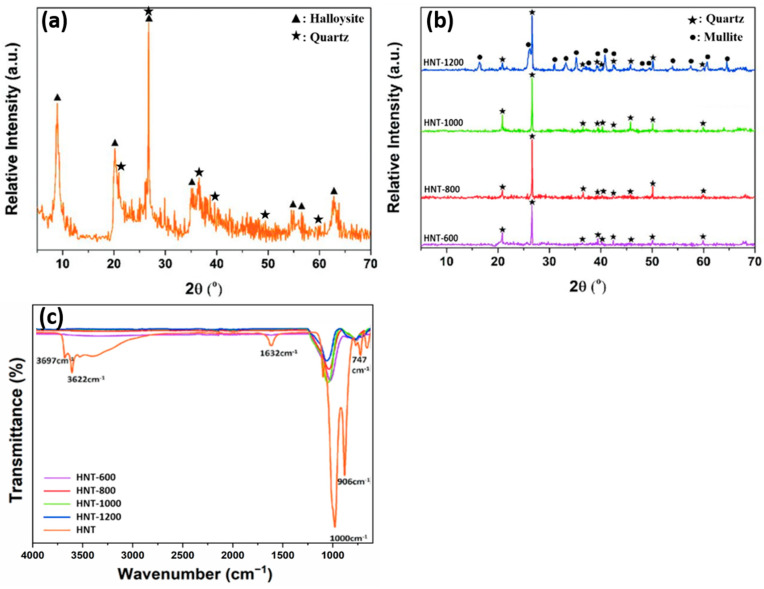
X-ray diffraction patterns of raw HNT (**a**) and calcined HNT (**b**). Fourier transform infrared spectra of the indicated HNT samples (**c**) [38]. The numbers (600, 800, 1000, and 1200) indicate the calcination temperature in °C.

HNT can be characterized using different methods. X-ray diffraction analysis of raw halloysite (Figure 4a,b) typically shows a sharp peak corresponding to the characteristic basal reflection (001) at ~8.96° (2θ), which is attributed to the hydrated halloysite (10 Å) form (ICDD #029-1489), as well as traces of quartz (ICDD #046-1045). The characteristic peaks of halloysite 10 Å are at 20.22, 26.74, and 35.06° (Figure 4a). After calcination from 600 to 1200 °C, the peak at 8.96 ° disappears due to conversion to dehydrated halloysite (halloysite 7 Å) (Figure 4b). After heat treatment at 600, 800, and 1000 °C, in all halloysite samples, quartz is observed in pristine form, and the halloysite crystalline phase is converted into an amorphous phase through dihydroxylation [37]. Finally, when the temperature reaches 1200 °C, recrystallization occurs, and the mullite diffraction peak (ICDD # 015-0776) appears next to the quartz peak (Figure 4b) [28,38]. 

Fourier transform infrared spectroscopy of raw halloysite (Figure 4c) highlights the presence of well-defined hydroxyl stretching bands that are typical of kaolin minerals at 3700–3600 cm^−1^. Raw and calcined halloysite samples show bands related to Si-O stretching in the 1000 cm^−1^ region. The band at 1632 cm^−1^ corresponds to strong bending vibrations of the adsorbed water [39]. After calcination, band intensity decreases to 1000 cm^−1^ compared with HNT. Upon calcination at 600 °C, halloysite is dehydroxylated and metahalloysite is formed, leading to the disappearance of the O-H stretch bands of the internal hydroxyl groups and internal surface hydroxyl groups (3622 cm^−1^ and 3697 cm^−1^, respectively). The peaks at 999.76 cm^−1^, 906.67 cm^−1^, and 748.43 cm^−1^ correspond to Si-O stretching and O-H deformation vibration of the internal hydroxyl groups [20,38,40,41,42,43].

**Figure 5:**
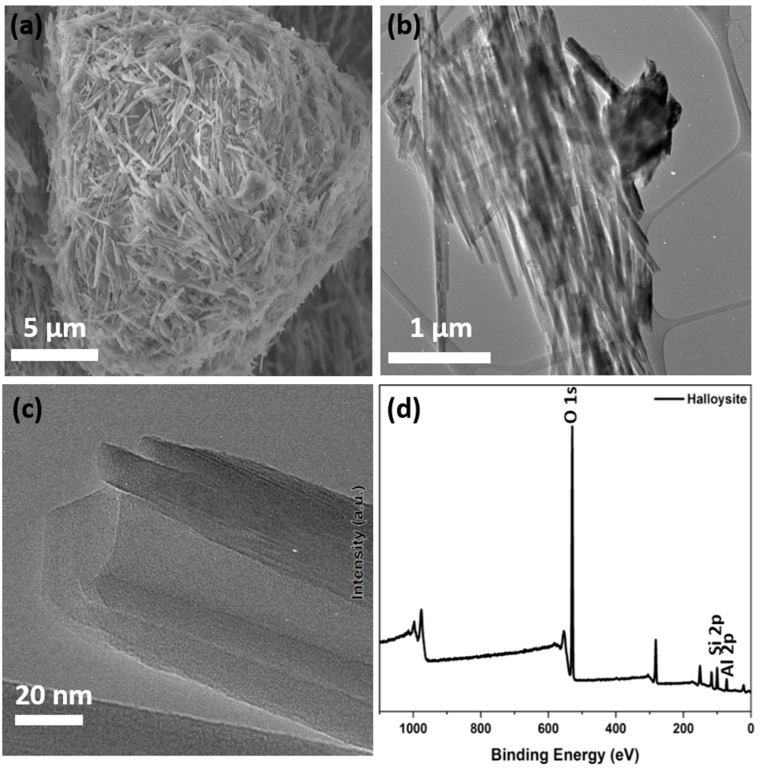
Morphological characterization by scanning electron microscopy (**a**) and transmission electron microscopy images (**b**,**c**), and X-ray photoelectron spectroscopy survey (**d**) of raw HNT from Tamra, Tunisia [41].

The morphological analysis by scanning electron microscopy (Figure 5a) shows tubular structures with cylindrical and prismatic HNT with a diameter between 50 and 70 nm [41]. Moreover, transmission electron microscopy (Figure 5b,c) highlights the presence of a nanotubular structure with outer and inner diameters of 60 ± 2 nm and 15 ± 5 nm, respectively [31,41]. X-ray photoelectron spectroscopy, which is used to determine the surface structure and chemical state of each element (Figure 5d), shows the presence of O 1s, Al 2p (indicating the presence of Al^3+^), and Si 2p (demonstrating the presence of SiO_2_) [41].

Zeta potential measurements show that the groups present on the outer surface of HNT (composed of SiO_2_-bonded layers) contribute to the negative zeta potential, and the inner cavity (consisting of layers with Al_2_O_3_) has a strong positive charge [27,31,32,34]. 

Many researchers have studied HNT from a theoretical viewpoint due to their intriguing properties and have tried to generate computational models of these nanotubes [44,45,46]. For instance, Guimarães et al. investigated the electronic and mechanical properties and also the stability of single-walled HNT 39. It is worth noting that they did not take into account any interlayer water molecules. However, theoretical research on this topic has been limited, and many studies have focused on the interactions between water molecules and other clay materials, particularly those with flat structures (e.g., kaolinite or dickite) [47,48]. 

## 3. Extraction and Purification of Natural Halloysite 

Natural halloysite clay contains impurities (e.g., kaolin, illite, quartz, feldspar, chlorite, gibbsite, salts, and metals) that greatly influence the nanotube size distribution. Therefore, different purification methods (see below) have been investigated to allow its subsequent modification and use for various applications [49,50,51]. 

### 3.1. Sedimentation and Purification 

Abid et al. dispersed natural halloysite in deionized water and, after overnight sedimentation, removed the fraction with a diameter >2 µm. They washed the obtained clay five times with deionized water and NaCl, followed by centrifugation. Then, they washed Na^+^ HNT with distilled water and centrifuged them until the test in the presence of silver nitrate (AgNO_3_) was negative. They dried the purified HNT in an oven at 110 °C for 3 h, followed by crushing and sieving [41,51].

### 3.2. Base-Treated Purification

Zhang et al. described a base-treated purification method that does not require high temperatures. Briefly, they mixed crude halloysite and water and then adjusted the pH to alkaline. After adding a dispersing agent, they stirred the solution at room temperature for 6 h. Finally, they collected the purified HNT by centrifugation or filtration [52].

### 3.3. Drying and Ball Milling

Sakiewicz and Lutynski dried HNTs at 60 °C and crushed them into particles of a size <10 mm. After ball milling for 20 min, they immersed 400 g of the obtained material in 550 cm^3^ of water. After stirring and washing, they ground the resulting slurry in a ball mill with steel balls 1–5 mm in diameter. Finally, they washed the material for 4 h [53].

### 3.4. Magnetic Separation 

To eliminate iron-containing impurities, Sakiewicz et al. proposed a multi-gradient magnetic separation approach to separate aluminum ferric silicate in weak magnetic field conditions [54]. With this method, they could remove heavy magnetic minerals (e.g., Fe_3_O_4_) that are usually difficult to eliminate. Alternatively, they suggested treating halloysite with hydrochloric acid, which will also remove other metal oxide impurities (e.g., copper, calcium, and titanium oxides) to further improve purification [50]. 

### 3.5. Acid Leaching and Ball Milling

Sakiewicz and Lutynski used sulfuric acid leaching to improve purification. After drying at 100 °C for 1 h, they used ball milling to crush the raw material, which was then homogenized, stirred, and washed. This was followed by ball milling (steel balls of 5 to 10 mm in diameter) and washing. Then, they added H_2_SO_4_ to the slurry for leaching in a reactor at 90 °C for 90 min. After washing and filtering the material to separate the magnetite and non-magnetic fractions, they used slow magnetic separation. Then, they separated the particle fraction with a size <20 µm in a sedimentation column. After filtering and drying, the material was ready for microscope analysis [53]. 

### 3.6. Ultrasonic Dispersion

Rong et al. used ultrasound to enhance halloysite dispersibility in the aqueous phase. They sonicated the suspension with an ultrasonic cell disruptor at 100–700 W for 100–1500 s. After sonication, they centrifuged the halloysite suspension in an ultracentrifuge. After discarding the supernatant, they collected the resulting sediments and washed them alternately with water and ethanol (three times/each). Finally, they dried the sediments at 60 °C for 12 h [55].

## 4. Modification of Halloysite Outer and Inner Surfaces 

**Figure 6:**
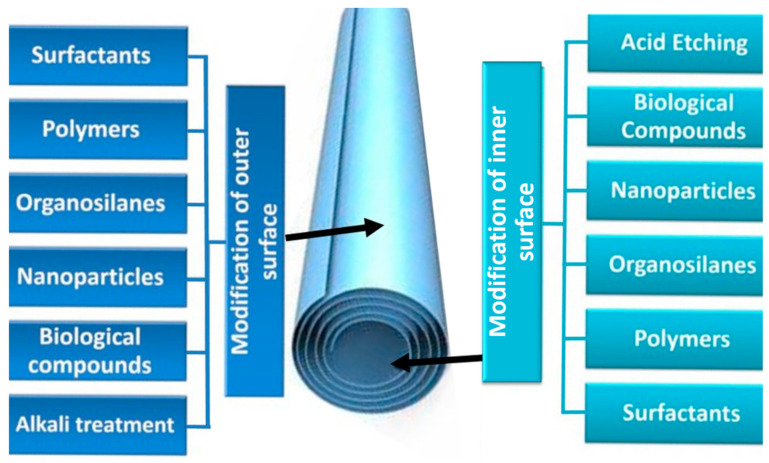
HNT outer and inner surface modifications.

The selective modifications of the surface properties summarized in Figure 6 highlight the great application potential of halloysite. The term “surface modification” describes the introduction of functional groups to modify/enhance specific physicochemical features (e.g., hydrophilicity/hydrophobicity, dispersion, rheology, reactivity, biotoxicity, electrochemistry). 

The siloxane (Si-O-Si), aluminol (Al-OH), and silanol (Si-OH) groups present on the HNT outer surface facilitate bonding with different functional groups through van der Waals forces, hydrogen bonding, electrostatic, and covalent interactions [56]. This allows immobilizing different molecules on the HNT outer surface to modify it, without affecting the nanotube structure. Different surface modifications can be used depending on the chosen application (see list in Table 1).

Alkali treatment (often conducted with sodium hydroxide (NaOH)) reduces HNT wall thickness and increases the surface hydroxyl density. The final result is influenced by the experimental conditions (e.g., alkali concentration, stirring speed). When the lumen has a large diameter for efficient loading or encapsulation of various functional compounds, increasing the HNT wall thinning and hydroxylation improves HNT stability and reactivity in aqueous solutions. In the presence of water molecules, the siloxane units on the HNT surface form silanol groups. NaOH dissociation with Na^+^ and OH^−^ also plays a crucial role in the deprotonation of such silanol groups [57].

HNT can also be modified with polymers and biopolymers through non-covalent bonding to increase their strength and antibacterial, thermal, and electrical activities. Moreover, functional groups can be attached to the HNT surface using polymers and biopolymers to increase the SSA and reactivity, particularly for drug delivery.

Indeed, after surface modification (also called first-layer modification), HNT can be used for the loading and release of different drugs (e.g., non-steroidal anti-inflammatory drugs, antibiotics), enzymes, and nanoparticles. For instance, HNT outer surface can be modified using organosilanes that display low toxicity [e.g., 3-aminopropyltriethoxysilane, 3-(2-aminoethylamino)propyltrimethoxysilane, 3-mercaptopropyltrimethoxysilane]. Organosilanes are frequently chosen because they harbor different organofunctional groups that enhance the binding of other molecules (e.g., drugs) to the surface. Moreover, HNT functionalization limits the complexation of antibiotics with metal ions by forming an amine-metal ion-amine bond, thus increasing their bioavailability [58].

HNT’s outer surface may also be modified with plant-derived compounds, such as polyphenols. Polyphenols (e.g., tannic acid) are green and cheap materials with a large number of gallol groups to efficiently bind to any solid-liquid interface. Tannic acid-modified HNTs have been used to produce nanofiltration membranes with higher hydrophilicity and surface charge [57].

Nanoparticles can also be used to modify HNT directly (nanoparticle immobilization on HNT’s pristine surface) or after a first modification. This functionalization increases HNT conductivity and catalytic activity. Many studies have described HNT outer surface modification with metal (e.g., Ag, Au, and Ru) and metal oxide nanoparticles (e.g., cobalt, iron, and titanium oxide) [7,59,60,61,62] (described in detail below).

HNT can also be modified with surfactants (i.e., organic compounds with hydrophobic and hydrophilic parts) to reduce the tension between the HNT surface and water molecules. This improves HNT dispersibility in nonpolar solutions and limits aggregation. Moreover, this modification favors the formation of micelles, a particularly interesting feature when HNTs are used as carriers of functional compounds. Such micelles can be exploited to encapsulate aliphatic hydrocarbons for pollution remediation. The choice of surfactant type is based on the HNT surface area (negatively charged). Cationic surfactants create water-in-oil emulsions in which antimicrobial agents are encapsulated in reverse micelles generated on the HNT surface. Reverse micelles are formed when the surfactant’s positive head groups are adsorbed, followed by the formation of nanoparticles with a hydrophilic core and a hydrophobic shell. Surfactants are also used to decrease the HNT surface hydrophilicity. For instance, Feng et al. used n-hexadecyltrimethoxysilane and tetraethoxysilane (cationic surfactants) to modify the HNT surface into a very hydrophobic surface. Upon the formation of the poly-siloxane network, the HNT surface changed from smooth to rough. This modification should reduce HNT transparency and increase their water repellency.

**Table 1:** Halloysite nanotube outer and inner surface modifications.

Modification	Category	Example	Applications	Ref
Outer surface	Alkali etching	NaOH	HNT toughening	[57]
	Biological compounds	Tannic acid	Dye and salt separation	[63]
	Nanoparticles	Ru	Preferential oxidation of CO in H_2_	[59]
		Co_3_O_4_	Enhancement of magnetic properties	[7]
		Fe_3_O_4_	Synthesis of dihydropyrimidinones	[62]
		TiO_2_	Photocatalysis	[64]
		ZnO	Antibacterial activity	[61]
		Au	Benzyl alcohol oxidation	[60]
		Ag	Antibacterial activity	[61]
	Organosilanes	3-aminopropyl-triethoxysilane (APTES)	Immobilization of α-amylase	[65]
		3-(2-aminoethylamino)propyltrimethoxysilane (AEAPTMS)	Antibacterial activity	[66]
		3-mercaptopropyltrimethoxysilane (MPTS)	New drug delivery system	[67]
	Polymers and biopolymers	Poly(3,4-ethylenedioxythiophene) PEDOT	-	[68]
		Polyethylenimine (PEI)	Gene delivery	[34]
		Polyaniline	Sensitive ascorbic acid sensor	[69]
		Pectin	Adsorption	[70]
		Hexadecyltrimethoxysilane (DNA)	-	[71]
		Polydopamine	Catalyst for ammonia borane hydrolysis	[72]
		Chitosan	Drug carrier	[73]
	Surfactants	Alkyltrimethyl ammonium bromide	Reverse micelles for water-in-oil emulsion	[74]
		Hexadecyltrimethoxysilane (HDTMS)	Oil water separation	[75]
Inner surface	Acid etching	H_2_SO_4_	-	[76]
		HCl	Adsorption and release of ofloxacin	[77]
	Biomolecule	Curcumin	Adsorption	[78]
	Nanomaterials	Ag	Photocatalysis	[79]
		Cu-Ni	-	[80]
		Carbon nanodots	-	[81]
	Organosilanes	APTES	Adsorption	[82]
	Polymers	Polymethyl methacrylate (PMMA)	-	[83]
		Polyethylene oxide (PEO)	-	[84]
	Surfactant	Perfluoropentanoic acid (PCF5H) Perfluoroheptanoic acid (PCF7H) Perfluorooctanoic acid (PCF8H)	-	[85]

## 5. Nanocomposites Based on HNT and Semiconductors 

HNTs have a 1D tubular morphology, are abundant, and are cheaper than other nanotubes (e.g., carbon nanotubes). Notably, their crystal structure has an aspect ratio similar to that of carbon nanotubes. Moreover, HNTs are characterized by an ordered structure with aluminum alcohol groups attached to the inner surface and silanol groups on the outer surface. Similar to other inorganic nanotubes, HNTs are insulators and rigid without enhanced electron transfer. HNT structures and properties can be modified to use them as catalysts with good adsorption, electronic conductivity, and thermal stability. Moreover, to increase their affinity and loading capacity, HNT can be decorated with semiconductors due to their layered structure. Many researchers have shown that HNT-semiconductor composites are low-tech, cost-effective alternative nanomaterials with superior mechanical, thermal, and biological properties. Table 2 summarizes various HNT applications, especially adsorption, heavy metal removal, and photocatalysis, depending essentially on the band gap, surface area, and electron–hole pair generation [41].

Li et al. produced a graphitic carbon nitride (g-C_3_N_4_)-ZnO/HNT nanocomposite photocatalyst using calcination to increase the visible-light photocatalytic activity and stability of ZnO photocatalysts. They showed that such a composite photocatalyst was more photo-responsive and more stable compared with ZnO/HNT. Halloysite strengthened the charge transfer pathways and prolonged the photoexcited carrier lifetime by increasing the surface area of g-C_3_N_4_ [86]. Shu et al. integrated HNT and silver nanoparticles into ZnO nanoparticles to produce an antimicrobial nanocomposite. HNT increased ZnO nanoparticle dispersion and stability and facilitated their contact with *Escherichia coli (E. coli)*. Moreover, the silver nanoparticles favored the separation of photogenerated electron–hole pairs and increased the ZnO nanoparticle antibacterial activity and stability [61]. Similarly, Shu et al. found that CeO_2_-ZnO/HNT antibacterial activity against *E. coli* was increased compared to ZnO alone. This was attributed to the reduced ZnO agglomeration mediated by HNT as well as the synergetic effect of CeO_2_, which inhibits the fast recombination of hole–electron pairs and reduces the energy gap of ZnO [87]. 

Zhang et al. fabricated metal oxide/carbon-coated HNT nanocomposites in which the synthesis of carbon-coated HNT was followed by the deposition of ZnO and TiO_2_ nanoparticles. Their good photocatalytic performance for the photodegradation of methylene blue dye indicated that carbon coating enhances HNT electron conductivity and charge–carrier separation in metal oxide semiconductors [88]. 

**Figure 7:**
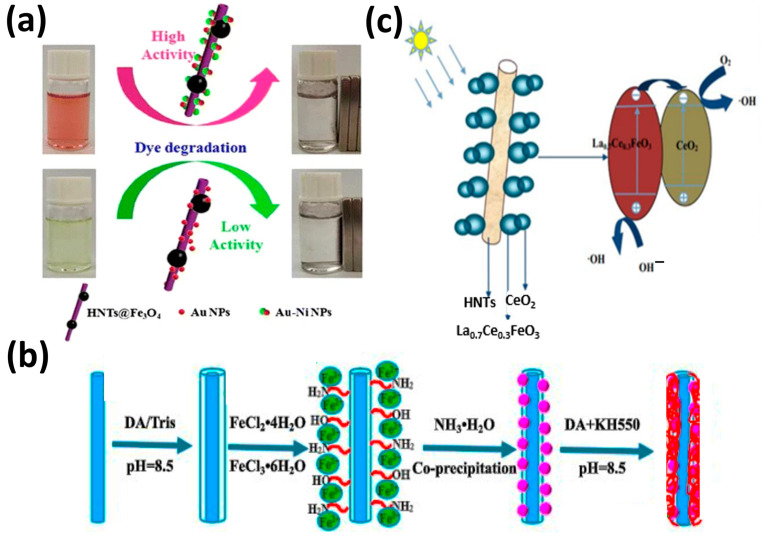
Photocatalytic degradation of organic dyes in the presence of HNT@Fe_3_O_4_@Au and HNT@Fe_3_O_4_@Au–Ni [90] (**a**). Description of the HNT/Fe_3_O_4_/poly(DA + KH550) nano-hybrid adsorbent [91] (**b**). Mechanism of photocatalysis in the presence of La_0.7_Ce_0.3_FeO_3_/HNT [92] (**c**).

Rostami et al. produced zinc ferrite–graphene nanohybrids with different amounts of graphene. They found that zinc ferrite nanopowders were inactive under visible irradiation, as indicated by the absence of paracetamol degradation. The addition of 4% graphene led to the complete degradation of paracetamol after 3 h of visible irradiation [89]. Jia et al. fabricated two multicomponent nanocatalysts (HNT@Fe_3_O_4_@Au and HNT@Fe_3_O_4_@Au–Ni) using hydrophilic magnetic HNT and symmetric or Janus nanoparticles (Figure 7a). Investigation of the reduction in Congo red and 4-nitrophenol with NaBH4 by these two nanocatalysts revealed that the catalytic activity of the nanocatalyst decorated with Au–Ni nanoparticles was much higher than that of the isotropic nanocatalyst decorated with Au nanoparticles. This showed the important role of Janus Au–Ni nanoparticles in these reactions [90].

**Figure 8:**
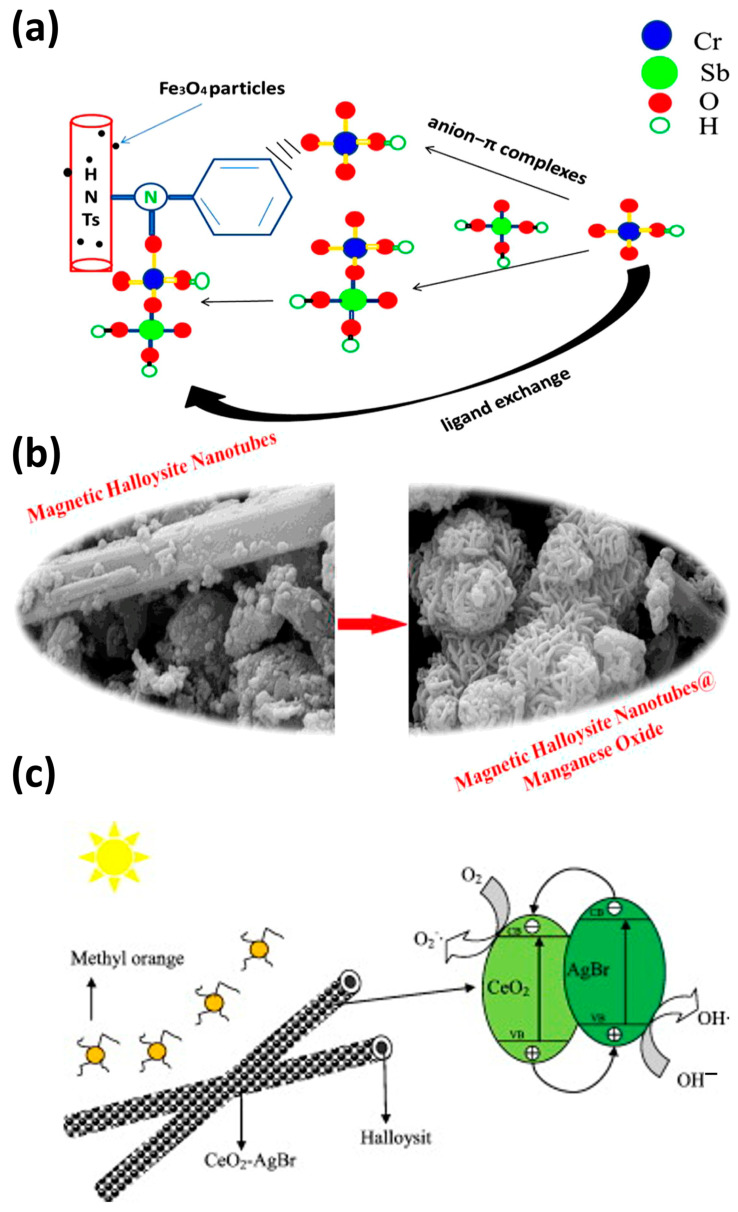
Schematic representation of the concomitant removal of Cr(VI) and Sb(V) in the presence of silane-modified HNT/Fe_3_O_4_ nanocomposites [93] (**a**). MnO_2_ nanoparticle deposition on magnetic HNT using the hydrothermal method [96] (**b**). HNT-CeO_2_-AgBr photocatalyst [102] (**c**).

Wan et al. described a new core@double-shell structured HNT/Fe_3_O_4_/poly(dopamine + hydrochloride-triethoxysilane (DA + KH550)) nano-hybrid adsorbent for removing methylene blue (Figure 7b). HNT/Fe_3_O_4_/poly(DA + KH550) showed high methylene blue adsorption capacity from an aqueous solution (714.29 mg/g) and very good stability [91]. Li et al. prepared (sol–gel method) hierarchical La_0.7_Ce_0.3_FeO_3_/HNT composites (Figure 7c). These composites showed very good photocatalytic activity and removed 99% of chlortetracycline under visible light irradiation for 90 min. This good performance was explained by HNT adsorptive capacity and the increased electron transfer capacity of La_0.7_Ce_0.3_FeO_3_ [92]. Zhu et al. fabricated (in situ growth) Fe_3_O_4_ nanoparticles on HNT that were then modified with different silane coupling agents (Figure 8a). The HNT/Fe_3_O_4_ composite modified with anilino-methyl-triethoxysilane (KH-42) adsorbed 100% of Cr(VI), 67% of Sb(V), and 98.9% of both Cr(VI) and Sb(V) when in the same solution. The authors proposed that Cr(VI) is responsible for the synergistic effect of the simultaneous adsorption [93]. Amjadi et al. prepared a HNT-Fe_3_O_4_ magnetic nanocomposite to be used as an adsorbent for solid-phase extraction and preconcentration of Cd(II) as Cd-phenanthroline complexes. This cheap nanocomposite, made using natural materials, simplifies Cd(II) extraction and is reusable. It could be used to detect Cd(II) traces in spiked waters, nails, and hair samples [94]. 

Xing et al. described a new cadmium sulfite/HNT photocatalyst (hydrothermal synthesis) that degraded 93% of tetracycline in 60 min under visible light [95]. Afzali and Fayazi used a magnetic HNT@MnO_2_ nanocomposite (hydrothermal synthesis) as a sorbent for the efficient removal of Pb(II) ions from aqueous solutions. This nanocomposite was stable and reusable (Figure 8b) [96].

Saraji et al. modified HNT (MHNT) by etching, hydroxylation, and amino-grafting. Then, they used the sol–gel technique to bond MHNT to a fused silica support. They tested the newly obtained sorbent material as a solid-phase microextraction (SPME) coating with very good thermal stability and durability. They found that for diazinon, parathion, and fenthion detection, SPME-MHNT displayed better extraction efficiency than commercial and homemade fibers (polyamide, polydimethylsiloxane, and polydimethylsiloxane/divinylbenzene). Then, they used SPME-MHNT to detect organophosphorus pesticides in agricultural wastewater, cucumbers, and apples without any significant matrix effect [97]. Fizir et al. synthesized HNT-based molecularly imprinted polymers (HNT@MIPs) by surface-initiated precipitation polymerization and computer simulation. They showed that HNT@MIPs are excellent sorbents for pollutant removal and could be used as biocompatible carriers for drug delivery [98]. 

Several groups demonstrated that HNT can be used as support to limit TiO_2_ agglomeration and increase the SSA (and consequently the absorbance and catalytic activity). Wang et al. showed that TiO_2_/HNT (31.8 wt% TiO_2_) has improved photocatalytic degradation performance (methanol and acetic acid) compared with TiO_2_ anatase [64]. Similarly, Papoulis et al. found that the decomposition of NOx gas and toluene is enhanced when TiO_2_ nanoparticles are deposited on HNT, compared with commercial Titania P25 [40]. Du et al. reported that a TiO_2_-HNT composite fabricated with the sol–gel method degraded 81.6% of methylene blue after 4 h of UV irradiation [25]. Zheng et al. showed that an amylose-HNT-TiO_2_ composite with high dispersion and a large surface-specific area (408.8 m^2^/g) removed 91% of methylene blue after 10 h of UV irradiation [99]. Moslehyani et al. fabricated nanofibrous adsorptive membranes by electrospinning nanofibrous nanoparticles with HNT-TiO_2_ nanoparticles (SSA of 17.9 m^2^/g) that removed 31.2 mg/g arsenic from contaminated water [100]. Jiang et al. used electrospinning to incorporate HNT into TiO_2_/HNT hybrid nanofibers. They obtained the best degradation performance (81% of methylene blue was eliminated after 90 min under visible irradiation) with the sample that included 8% HNT. They proposed that this positive effect was due to the mass transport of reactants into the active nanofiber centers [31]. 

Li et al. synthesized heterogeneous polyaniline-crystalline TiO_2_-HNT by modulating the acid dopant in the preparation. They obtained the highest photocatalyst activity (rhodamine B degradation in an aqueous solution) at pH 0.5 and 1% *v*/*v* of aniline and titanium isopropoxide. This showed that the photocatalytic activity can be improved by tuning the concentration and type of acid dopant [101]. Du and Zheng fabricated TiO_2_-HNT composites by depositing anatase TiO_2_ onto the HNT surface, followed by calcination at different temperatures. They found that by increasing the calcination temperature from 100 to 500 °C, methylene blue maximum adsorption increased from 38.57 to 54.29 mg/g [25]. Wu et al. showed that g-C_3_N_4_-TiO_2_/HNT composites (sol–gel and calcination) display higher photocatalytic activity under visible light (ciprofloxacin degradation) compared with TiO_2_/HNT. This effect may be attributed to the heterojunction structure between g-C_3_N_4_ and TiO_2_/HNT and to HNT introduction, which accelerates photoelectron–hole pair transfer and separation [86]. Li et al. synthesized halloysite-CeO_2_-AgBr nanocomposites (Figure 8c) that degraded 99% of methyl orange in 80 min. They proposed that their good stability after eight runs can be attributed to the presence of HNT and the g-C_3_N_4_-TiO_2_ heterojunction [102]. Papoulis et al. prepared two nanocomposites (palygorskite-TiO_2_ and halloysite-TiO_2_) using the sol–gel method followed by hydrothermal treatment at 180 °C. NOx gas decomposition under visible and UV light irradiation was increased when halloysite-TiO_2_ samples were used compared with titania P25. According to the authors, this effect was due to the presence of homogeneously dispersed TiO_2_ on the HNT surface. Their combination could be an interesting strategy to improve the photocatalytic degradation of pollutants [103].

**Table 2:** Metal-based semiconductors on halloysite nanotubes used for different applications.

Nanocomposite	Synthesis Method	Applications	Ref
Ag-ZnO/HNT	Sol–gel	*E. coli* antibacterial activity	[61]
CeO_2_-ZnO/HNT	Precipitation in ethanol system	*E. coli* antibacterial activity	[87]
Polymer-Fe_3_O_4_-HNT	Surface-initiated precipitation polymerization + computer simulation	Delivery of a cationic drug (norfloxacin)	[98]
Core/shell HNT/Fe_3_O_4_	Co-precipitation and modified mussel-inspired co-modification route	Adsorption of methylene blue	[91]
KH42@Fe_3_O_4_/HNT	In situ growth of Fe_3_O_4_ nanoparticles	Removal of heavy metals [Cr(VI), Sb(V)]	[93]
Fe_3_O_4_-HNT	Chemical precipitation	Removal of cadmium(II)	[94]
MHNT@MnO_2_	Precipitation and hydrothermal method	Lead(II) removal from aqueous solutions	[96]
HNT-TiO_2_-(solid phase microextraction)	Etching, hydroxylation, amino grafting, and sol–gel	Parathion removal	[97]
g-C_3_N_4_-ZnO/Hal	Calcination	Degradation of tetracycline under visible light irradiation	[86]
g-C_3_N_4_/TiO_2_/Hal	Sol–gel + calcination	Visible light photodegradation of ciprofloxacin	[104]
CeO_2_/AgBr-HNT	Microwave	Degradation of methyl orange	[102]
CdS-HNT	Hydrothermal	Degradation of tetracycline	[95]
Au–Ni/Fe_3_O_4_-HNT	Impregnation	Degradation of Congo red	[90]
Polyaniline–crystalline TiO_2_-HNT	Impregnation	Degradation of rhodamine B	[101]
TiO_2_-HNT	Impregnation	Degradation of methylene blue	[25]
ZnO or TiO_2_-HNT	Deposition	Degradation of methylene blue	[88]
LaFeO_3_-HNT	Sol–gel	Degradation of chlortetracycline	[92]
TiO_2_/Halloysite	TiO_2_ sol dispersion and hydrothermal treatment	Decomposition of NO_x_ gases	[103]

## 6. Heterogeneous Photocatalysis 

Chemical compounds such as hydrocarbons, phenolic compounds, heavy metals, dyes, pharmaceuticals, and insecticides that are present in wastewater cannot be easily biodegraded. This leads to water pollution and often results in their persistence in the environment [105,106,107,108,109,110].

Various methods have proven their effectiveness for removing persistent compounds in water, such as reverse osmosis, nanofiltration, adsorption on granular activated carbon, and advanced oxidation processes (AOP). AOP are a relevant strategy to tackle the water crisis. Currently, AOP are employed for the pre-treatment and/or post-treatment of conventional wastewater treatment methods. To enhance the generation of OH radicals, various catalysts are added during AOP to eliminate pollutants. Much research has focused on two AOP technologies: homogeneous photo-Fenton and heterogeneous photocatalytic processes.

**Figure 9:**
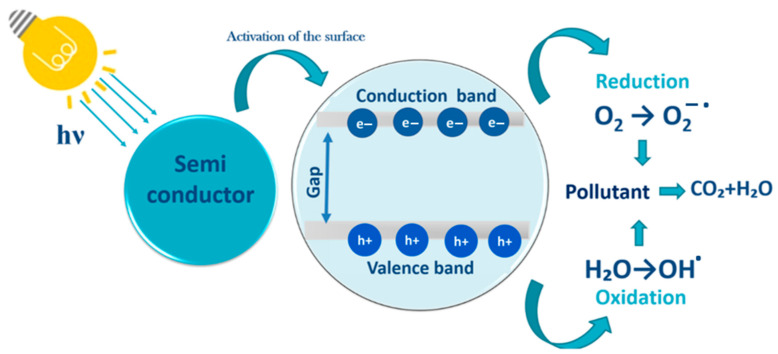
Schematic representation of the photocatalytic degradation of pollutants.

Heterogeneous photocatalysis is the most promising AOP for wastewater treatment. This efficient, cheap, and environmentally friendly technology [111,112,113] (Figure 9) is based on the activation of a semiconductor under light irradiation [114]. This leads to the excitation and transfer of electrons from the valence band to the conduction band and to the formation of electron–hole pairs that generate reactive species. Because of their high oxidizing capacity, these radicals allow the mineralization of the organic pollutants adsorbed on the photocatalyst surface and their transformation into H_2_O, CO_2_, and other mineral compounds (e.g., Cl^−^, SO_4_^2−^) [115,116].

Several semiconductors, such as ZnO, TiO_2_, g-C_3_N_4_, vanadium oxide (V_2_O_5_), and tin oxide (SnO_2_), have been evaluated for removing organic pollutants [117,118,119,120]. Among the semiconductors used for photocatalytic degradation, TiO_2_, especially in the form of anatase, is considered the most promising semiconductor oxide based on its good activity, stability, and low cost [121,122]. However, TiO_2_ absorbs only 3–5% of sunlight (UV radiation) due to the anatase large band gap [123,124] and displays limited recovery after utilization and fast recombination of the photogenerated charges [125,126]. Moreover, from an energetic point of view, a material with photocatalytic activity only under UV irradiation is not profitable. Therefore, it is important to develop economically interesting photocatalysts that can be activated by radiation in the visible spectrum, green, abundant, and free energy [127]. Many groups have assessed TiO_2_ immobilization on an inert support (e.g., stainless steel) [128]. However, this decreases TiO_2_ photocatalysis efficiency due to the reduced SSA of the irradiated photocatalysts [129]. Some studies investigated porous materials (e.g., silica gel, zeolites, and clays) as supports for pollutant adsorption [130,131,132]. Fibrous minerals, such as palygorskite, sepiolite, and halloysite are particularly interesting due to their ability to adsorb and retain pollutants, as well as their cationic exchange properties and the presence of silanol groups, which allow for structural modification [41,133,134].

Due to its versatile features, halloysite is a promising support for assembling nanoarchitectures for pollutant photodegradation. Indeed, HNT allows for improving TiO_2_ nanoparticle and nanofiber dispersibility and photocatalytic activity, limiting TiO_2_ agglomeration, and producing adsorbents and catalysts with higher SSA [135,136].

## 7. Halloysite-TiO_2_ Nanocomposites for Water Treatment

Recently, clay has been used to eliminate toxic waste from wastewater. Among these remarkable adsorbents, kaolin shows outstanding adsorption and photocatalytic activity as a support material for semiconductors. Its layered structure and size are considered advantageous for developing non-toxic and low-cost photocatalysts with high catalytic activity and stability [137,138]. The theoretical formula of HNT, as rolled aluminosilicate sheets, is similar to that of kaolinite, with the presence of interlayer water molecules as a distinguishing feature compared to kaolinite [138].

Halloysite-TiO_2_ nanocomposites can be exploited for different applications, including drug delivery, polymer filler, matrix for photocatalysts, and adsorbents for environmental and biomedical applications. In this part of the review, we will describe the development of inexpensive and effective HNT-TiO_2_ nanocomposites with very high absorption capacity to eliminate inorganic and organic pollutants present in water.

**Figure 10:**
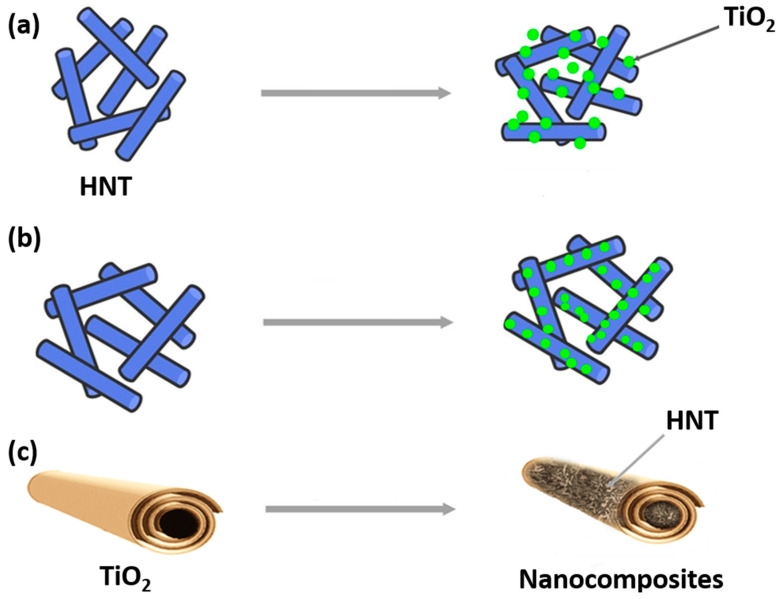
Schematic representation of HNT decorated with TiO_2_ (**a**), HNT loaded with TiO_2_ (**b**), and HNT loaded inside TiO_2_ nanofibers (**c**).

Recently, several studies have investigated the photodegradation of dyes, pesticides, and antibiotics present in water using either pristine or modified HNT decorated with TiO_2_ nanoparticles on their outer surface (Figure 10a) or loaded with TiO_2_ nanoparticles on their inner surface (Figure 10b). Additionally, some studies have explored the use of halloysite loaded inside TiO_2_ nanofibers for the same purpose (Figure 10c).

In the last few decades, due to the increase in industrial activities, water pollution has become a major health and environmental issue worldwide [139,140]. Halloysite-TiO_2_ nanocomposites present great potential for the removal of water contaminants due to their tubular structure, porous surface, low cost, environmental friendliness, and mechanical and chemical resistance (Table 3).

**Figure 11:**
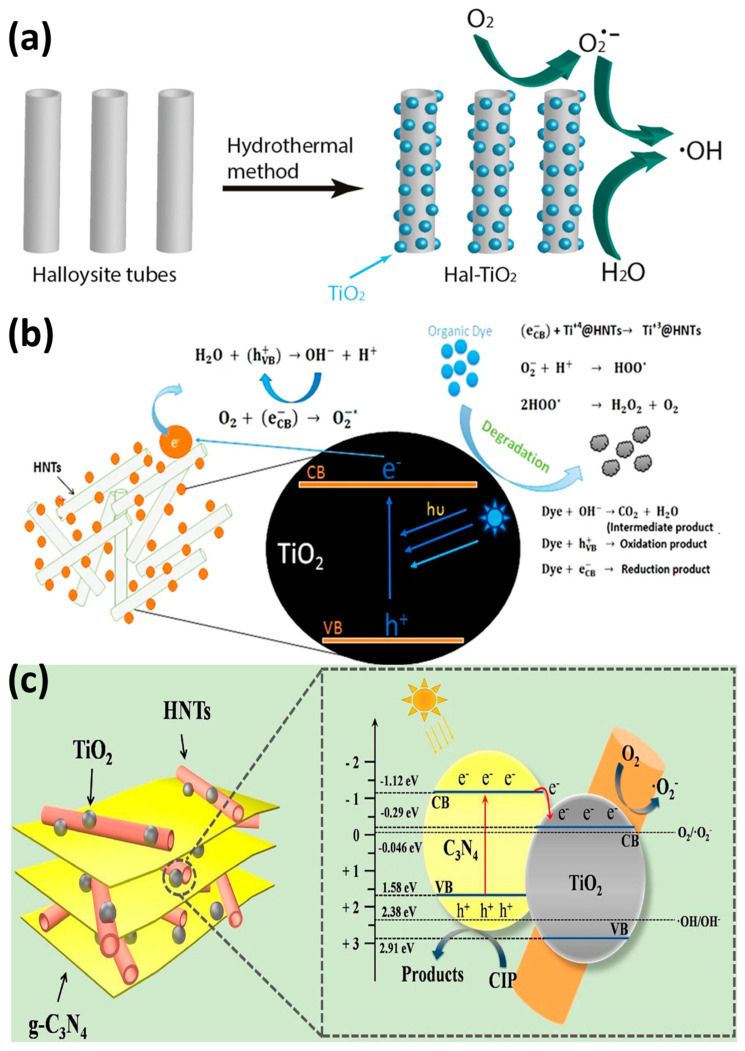
Schematic illustration of the production of the TiO_2_/HNT photocatalyst [42] (**a**). Schematic description of the photocatalytic activity of TiO_2_@HNT [136] (**b**). Ciprofloxacin (CIP) degradation in the presence of g-C_3_N_4_/TiO_2_/HNT [104] (**c**).

The nanocomposites that are shown in Figure 2 can be used to remove dyes from aqueous environments. Dyes are used mostly by the textile and paint industries and are removed by adsorption and/or degradation. Du and Zheng deposited anatase TiO_2_ on HNT surfaces using calcination at different temperatures (100 to 500 °C) to obtain TiO_2_-HNT composites. They found that at higher calcination temperatures, the anatase crystalline structure improved, but the HNT structure was damaged at the highest temperature. All tested samples showed very high adsorption capacities that ranged from 38.57 to 54.29 mg/g. The TiO_2_-HNT composite fabricated at 300 °C could eliminate 81.6% of methylene blue after 4 h of UV irradiation [25]. Rapsomanikis et al. fabricated TiO_2_/HNT thin films by adding silver using an acetic acid-based sol–gel method. The sample with 20–30% HNT showed higher photooxidation activity (tested by monitoring Basic Blue 41 azo dye degradation in water under UV irradiation) and better stability than Titania P25 [141]. Similarly, Papoulis et al. showed that the photocatalytic activity of TiO_2_/HNT (Figure 11a) and TiO_2_/HNT + sepiolite nanocomposites (fabricated with a hydrothermal method at 180 °C) was better than that of Titania P25 (paracetamol, tetracycline, and rhodamine B removal under UV visible light). They explained that this good result was due to the electrostatic attraction forces on the negatively charged HNT surface [42]. Yao et al. assessed the photocatalytic activity of amorphous C + N/TiO_2_/HNT with different mass ratios (fabricated using the precipitation-dissolution-recrystallization method and calcination at 550 °C for 4 h). After 1 h under natural light, C + N/TiO_2_/HNT with a mass ratio of 4.5 and TiO_2_/HNT degraded 95% and 85% of methylene blue, respectively. This difference was explained by the larger BET surface. C + N/TiO_2_/HNT remained stable after five cycles [142]. Li et al. synthesized 1D-polyaniline (PANI)-TiO_2_-HNT nanocomposites at different pHs and different volume concentrations with a low-temperature synthesis method. The samples prepared at pH 0.5 and with 1% *v*/*v* showed the highest photoactivity for rhodamine B degradation due to the PANI sensitizing effect and the charge transfer to TiO_2_. The PANI-TiO_2_-HNT photocatalyst degraded 73.49% of rhodamine B (10 mg/L) and was reused four times without loss of photoactivity under visible light. The authors propose that HNT can be used for wastewater treatment [143]. Mishra et al. fabricated a TiO_2_@HNT photocatalyst by combining sol–gel and phase inversion (Figure 11b). This photocatalyst displayed good stability and improved photocatalytic activity due to the electrostatic interaction between TiO_2_ and the HNT surface. The nanocomposite degraded 87.47% and 96.87% of 20 mg/g methylene blue and rhodamine B, respectively, under UV light [136]. Zheng et al. showed that amylose/HNT/TiO_2_ and HNT/TiO_2_ nanocomposites (fabricated by ball milling and sol–gel synthesis) have excellent catalytic activity and good stability for the removal of methylene blue under UV irradiation [99].

Pharmaceutical compounds, such as painkillers, anti-inflammatory drugs, antibiotics, and hormones, are discharged into sewage treatment plants and are detected in surface water, groundwater, and drinking water due to their incomplete elimination. Wu et al. evaluated new hetero-structural g-C_3_N_4_/TiO_2_/commercial halloysite composites (sol–gel and calcination at 500 °C) to eliminate ciprofloxacin from wastewater (Figure 11c). All composites (various mass ratios of melamine) displayed excellent photocatalytic activity under visible light and were stable. Particularly, the heterojunction composite eliminated 87% of ciprofloxacin in 60 min due to rapid photoelectron–hole pair transfer and separation [104]. Yu et al. prepared TiO_2_-Hal nanocomposites by introducing fly-ash cenospheres into HNT, followed by rare earth ion imprinting. Then, they assessed their photocatalytic activity by monitoring the degradation of 40 mg/L tetracycline under visible light irradiation. The as-prepared photocatalyst modified by the functional monomer o-phenylenediamine had the highest photocatalytic activity (78.80% of tetracycline degraded in 50 min) [144]. Wang et al. synthesized Ce-doped TiO_2_/HNT with a modified sol–gel method. By monitoring the photocatalytic degradation of tetracycline, they found that the photocatalytic performance of Ce-TiO_2_/HNT was increased by approximately seven times compared with that of TiO_2_/HNT. This improvement was due to the reduction in the distance between the conduction and valence bands of TiO_2_ by Ce doping [145]. 

**Figure 12:**
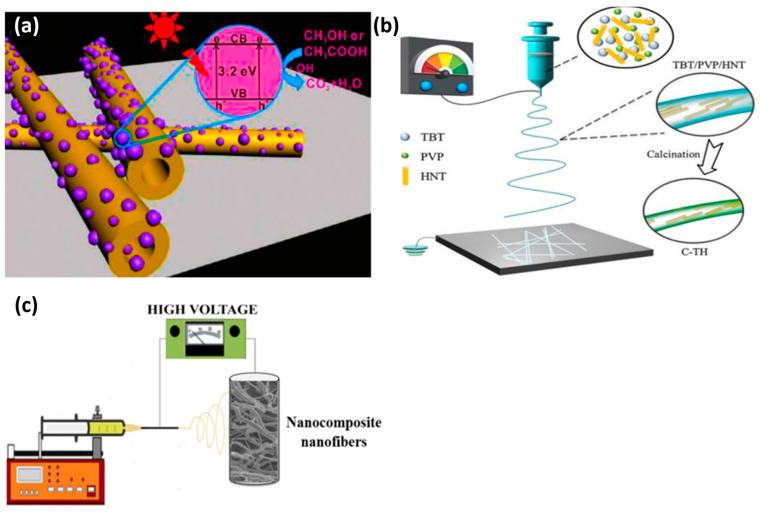
Photocatalytic process mediated by TiO_2_ deposited on HNT [147] (**a**). Carbon/TiO_2_/HNT hybrid nanofibers [31] (**b**). Illustration of the preparation of H95T5 nanofibers by combining halloysite and titanium oxide [41] (**c**).

Water contamination by pesticides from agricultural runoff entering nearby streams affects biodiversity, insects, birds, and other animal species. Szczepanik et al. used TiO_2_-halloysite composites for degrading chloroaniline in water by photocatalysis. The nanocomposites were produced using titanium isopropoxide as a precursor and the hydrothermal method at 65 °C. The surface area and pore volume were increased in acid-activated halloysite samples. The photocatalytic performance of these nanocomposites for the degradation of aniline, 2-chloro, and 2,6-dichloroaniline under UV irradiation was improved compared with commercial Titania P25 [146]. Wang et al. showed that TiO_2_/HNT (TiO_2_ deposition on HNT surface by the one-step solvothermal method) (Figure 12a) displays a pH-sensitive degradation performance, with higher photocatalytic activity for acetic acid degradation [64]. Panagiotaras et al. were the first to investigate pesticide decomposition using TiO_2_/HNT nanocomposites (fabricated with Hal nanotubes and the sol–gel method at 180 °C). They obtained the best performance with the halloysite-TiO_2_ (10–90%) nanomaterial: 47.4% of tebuconazole (a fungicide) degradation under UV and visible light irradiation versus 33.2% for Titania P25F [147]. 

Unlike the synthesis of TiO_2_-decorated halloysite, the synthesis of TiO_2_-HNT nanofibers is still in its infancy. Electrospinning is a simple method to produce nanofibers and fibers with different morphologies (e.g., hollow tubes, ribbons) [148] and with high production efficiency. Recently, Jiang et al. used the one-pot electrospinning/sol–gel method to fabricate carbon/TiO_2_/HNT (Figure 12b). By monitoring methylene blue degradation under visible light, they showed that adding a modest HNT amount (8%) increases the degradation performance by 23 times compared with commercial TiO_2_ anatase. This improvement was due to the improved mass transport of the reactant into the nanofibers [31]. Abid et al. used sol–gel and electrospinning to fabricate HNT-TiO_2_ nanocomposites (Figure 12c) for the degradation of acetaminophen and methylene blue under UV and visible light. The nanocomposite, known as H95T5, which comprised 95% natural halloysite and 5% TiO_2_, was able to degrade over 91% of acetaminophen and methylene blue after 150 and 360 min of exposure to visible light, respectively. They found that h^+^ and O_2_^−^ played a major role in photocatalysis [41].

**Table 3:** Degradation efficiency of different TiO_2_-halloysite nanocomposites.

Photocatalyst (g/L)	Method	Pollutant (mg/L)	Irradiation Type	Removal Efficiency (%)	Degradation Time (min)	Ref
Pani-TiO_2_-HNT (0.5 g/L)	Low temperature	Rhodamine B (10 mg/L)	XPA-7 photochemical system (800 W Xe lamp)	73.49	360	[143]
g-C_3_N_4_/TiO_2_/HNT (0.8 g/L)	Sol–gel and calcination	Ciprofloxacin (15 mg/L)	300 W Xe lamp	87.00	60	[104]
H95T5 (0.5 g/L)	Sol–gel + electrospinning	Acetaminophen (10 mg/L)	Medium-pressure metal halide UV	100	60	[41]
		Acetaminophen (10 mg/L)	Halogen linear lamp	91.10	360	
		Methylene blue (6.64 mg/L)	Medium-pressure metal halide UV	99.98	20	
		Methylene blue (6.64 mg/L)	Halogen linear lamp	96.83	180	
TiO_2_@HNT (4.2 g/L)	Sol–gel + Phase inversion	Methylene blue 20 mg/L	125 W UV lamp (254 nm)	87.47	120	[136]
		Methylene blue 20 mg/L		96.87	120	
TiO_2_/HNT (0.5 g/L)	Sol–gel	Methylene blue (32 mg/L)	12 W UV lamp (λ = 365 nm)	81.60	240	[25]
TiO_2_/HNT (20 mg)	Solvothermal	Acetic acid 5 mL of 5%	100 W high-pressure lamp	3488.63 μmol/g	60	[64]
		Methanol 5 mL of 5%		729.37 μmol/g	120	
Ce-TiO_2_/HNT (0.5 g/L)	Sol–gel	Tetracycline 20 mg/L	300 W Xe lamp (λ > 420)	78.00	60	[145]
Ag/TiO_2_-HNT (0.625 g/L)	Sol–gel	Basic blue 41 (12 mg/L)	Four black light fluorescent of 4 W	100	100	[141]
Carbon-TiO_2_-HNT (8%) (0.2 g/L)	Sol–gel + Electrospinning	Methylene blue (20 mg/L)	50 W UV lamps (k < 420 nm)	81.00	90	[31]
Amylose-HNT-TiO_2_ (1 g/L)	Ball milling + Sol–gel	4-nitrophenol (10 mg/L)	12 W UV lamp (λ = 253 nm)	90.00	240	[99]

## 8. Reaction Kinetics of Photocatalytic Pollutant Degradation Using Halloysite-TiO_2_ Nanocomposites as Photocatalysts 

In most cases, the kinetics of heterogeneous catalytic processes are explained using Langmuir–Hinshelwood kinetics. Indeed, experimental evidence has demonstrated that the photocatalytic degradation rate of many organic pollutants in the presence of TiO_2_ can be modeled with the Langmuir kinetic equation [149]:

(1) $$r=-\frac{dC}{dt}=\frac{k_{r}\;KC}{1+KC}$$

where *r* is the reaction rate (mg/L·min) that changes with the irradiation time *t*; *C* is the pollutant (in our case) concentration (mg/L); *k_r_* is the reaction rate constant; and *K* is the adsorption equilibrium constant of the pollutant on the photocatalyst (L/mg). The term *r* is expressed as the initial reaction rate (*r*_0_) as a function of the initial pollutant concentration (*C*_0_) or the adsorption equilibrium concentration of the pollutant in solution (*C_e_*). The parameters *k_r_* and *K* can be predicted using the linearized Equation (1):

(2) $$\frac{1}{r_{0}}=\frac{1}{k_{r}}+\frac{1}{k_{r}\;KC0}$$

When *C*_0_ is small, the first-order kinetics for the condition *KC*_0_ « 1 can be used, and Equation (1) can be reduced to an apparent first-order equation: [149,150].


(3) $$\ln\left(\frac{C_{0}}{C}\right)=kKt=k_{a}t$$

The slope of the linear regression plot of ln *C*_0_*/C* as a function of time corresponds to the apparent first-order rate constant *k_a_*. The organic pollutant photooxidation rate in the presence of TiO_2_ and under irradiation fits the Langmuir–Hinshelwood model if the reaction occurs: (a) between two adsorbed substances; (b) between a radical in solution and the adsorbed substrate; (c) between a radical linked to the surface and a substrate molecule in solution; and (d) between two substances in solution.

Substrate adsorption strongly influences the photocatalytic degradation rate [146,149,150].

Some kinetic constants from pseudo-first-order kinetic models of different experiments fit the Langmuir model because *R*^2^ ≈ 1 (listed in Table 4). HNT-TiO_2_ composites exhibit much higher catalytic efficiency and kinetics compared with other HNT nanocomposites.

## 9. Conclusions and Perspectives

HNTs are a good alternative to carbon nanotubes for many applications because they are non-toxic, amenable to large-scale production, and highly biocompatible. This review provides an overview of HNT’s main properties that are useful for the development of novel nanocomposites. The improved degradation of organic pollutants by photocatalysis in the presence of HNT/TiO_2_ nanocomposites as photocatalysts can be explained by their high SSA and adsorption capacities, large pore volumes, good stabilities, and good mechanical properties. HNTs are an interesting support for TiO_2_ due to their tubular form. Their association decreases TiO_2_ particle agglomeration and prevents nanoparticle release in the environment.

Many efforts are needed to improve water treatment technologies to overcome water scarcity. Currently, heterogeneous photocatalysis is not widely used for water treatment due to several technical challenges, including those related to photocatalytic systems, material synthesis, substrate selection, membrane reactors, and process scaling. Many studies on the degradation of organic and inorganic compounds using various catalysts to enhance photocatalytic activity have been published over the years. The photocatalytic activity of catalysts can be improved by the deposition of different materials (e.g., oxides, metals, and non-metals) on different substrate types. Furthermore, modifications can be envisaged to produce robust materials for wastewater treatment and large-scale production. Therefore, the long-term stability and mathematical modeling for catalyst optimization must be considered before scaling up.

HNT-TiO_2_ photocatalysts have been successfully used in the laboratory for water and wastewater treatment under visible light. These structures can be modified to enhance their catalytic activity by doping them with metals or non-metals and linking them to other semiconductors. Finding the ideal dopant concentration is crucial to improving carrier separation and conductivity as well as preventing the formation of recombination centers. Therefore, more research on the development of HNT-TiO_2_-based materials is essential to identify the functions required for their industrial application. Importantly, the catalyst’s performance can be modulated by choosing the right approach to coat or fabricate the catalyst. Among the many techniques to improve catalyst performance, atomic layer deposition has recently drawn much attention as an interesting approach to producing highly structured materials and controlling the coating thickness. It allows the deposition of different materials, such as oxides, metals, and non-metals, on different types of substrates. This method could be used to produce strong and long-term stable catalysts for wastewater treatment [151,152].

It would also be interesting to investigate how to use HNT-TiO_2_ nanocomposites for electrocatalytic degradation and mineralization of contaminants. However, for this, it is necessary to determine the optimal conditions to ensure that the process is effective and does not cause any additional harm to the environment. Peroxymonosulfate activation could be one of the potential methods to achieve this goal [153].

## Figures and Tables

**Table 4:** Kinetic efficiency of different TiO_2_-halloysite nanocomposites.

Photocatalyst (g/L)	Pollutant (mg/L)	Irradiation Type	*K_obs_* (min^−1^)	*K_obs_*/(%TiO_2_)	Ref
H95T5 (0.5 g/L)	Acetaminophen (10 mg/L)	UV	0.09719	1.9438	[41]
	Acetaminophen (10 mg/L)	Halogen linear lamp	0.00641	0.1282	
	Methylene blue (6.64 mg/L)		0.0175	0.3504	
	Methylene blue (6.64 mg/L)	UV	0.1714	3.4284	
TiO_2_@HNT (4.2 g/L)	Methylene blue 20 mg/L	125 W UV	0.0073	0.0075	[136]
	Rhodamine B 20 mg/L		0.0024	0.0025	
Ag/TiO_2_-HNT (0.625 g/L)	Basic blue 41 (12 mg/L)	Fluorescent black light, 4 W	0.0282	0.0471	[141]
Carbon-TiO_2_-HNT (0.2 g/L)	Methylene blue (20 mg/L)	50 W UV	0.0184	0.0200	[31]

## Data Availability

Not applicable.
